# Screening Diagnostic Candidates for Schistosomiasis from Tegument Proteins of Adult *Schistosoma japonicum* Using an Immunoproteomic Approach

**DOI:** 10.1371/journal.pntd.0003454

**Published:** 2015-02-23

**Authors:** Min Zhang, Zhiqiang Fu, Changjian Li, Yanhui Han, Xiaodan Cao, Hongxiao Han, Yantao Liu, Ke Lu, Yang Hong, Jiaojiao Lin

**Affiliations:** 1 Key Laboratory of Animal Parasitology, Ministry of Agriculture of China, Shanghai Veterinary Research Institute, Chinese Academy of Agricultural Sciences, Shanghai, China; 2 College of Animal Science and Technology, Henan University of Science and Technology, Luoyang, China; 3 College of Animal Science, Henan Institute of Science and Technology, Xinxiang, China; 4 Jiangsu Co-innovation Center for Prevention and Control of Important Animal Infectious Diseases and Zoonoses, Yangzhou, China; University of Nottingham, UNITED KINGDOM

## Abstract

**Background:**

Schistosomiasis is one of the world’s most prevalent zoonotic diseases and a serious worldwide public health problem. Since the tegument (TG) of *Schistosoma japonicum* is in direct contact with the host and induces a host immune response against infection, the identification of immune response target molecules in the schistosome TG is crucial for screening diagnostic antigens for this disease.

**Methodology/Principal Findings:**

In this study, an immunoproteomics approach used TG proteins as screening antigens to identify potential diagnostic molecules of *S*. *japonicum*. Ten spots corresponding to six proteins were identified that immunoreacted with sera from *S*. *japonicum*-infected rabbits but not sera from uninfected rabbits and their specific IgG antibody levels declined quickly after praziquantel treatment. Recombinant phosphoglycerate mutase (PGM) and UV excision repair protein RAD23 homolog B (RAD23) proteins were expressed and their diagnostic potential for schistosomiasis was evaluated and compared with schistosome soluble egg antigen (SEA) using ELISA. The results showed high sensitivity and specificity and low crossreactivity when rSjPGM-ELISA and rSjRAD23-ELISA were used to detect water buffalo schistosomiasis. Moreover, antibodies to rSjPGM and rSjRAD23 might be short-lived since they declined quickly after chemotherapy.

**Conclusion/Significance:**

Therefore, the two schistosome TG proteins SjPGM and SjRAD23 were identified as potential diagnostic markers for the disease. The two recombinant proteins might have the potential to evaluate the effectiveness of drug treatments and for distinguishing between current and past infection.

## Author Summary

As one of the world’s most prevalent zoonotic diseases, schistosomiasis remains a serious worldwide public health problem. An immunoproteomics approach was used to identify potential immune diagnostic molecules from *S*. *japonicum* TG proteins extracted from adult schistosomes. Probes were derived from sera collected from uninfected rabbits and rabbits at 2 and 6 weeks after infection with *S*. *japonicum* as well as praziquantel-treated rabbits. SjPGM and SjRAD23 were identified as potential diagnostic markers for the disease. High sensitivity and specificity and low crossreactivity were observed for rSjPGM-ELISA and rSjRAD23-ELISA, which were used to detect water buffalo schistosomiasis. In addition, these two proteins induced a short-lived antibody response since specific IgG antibodies to them declined quickly after chemotherapy and had the potential to be used to evaluate the effectiveness of chemotherapy against *S*. *japonicum*. Our study provides information for screening potential diagnostic antigens and establishing more sensitive schistosomiasis diagnosis techniques.

## Introduction

Schistosomiasis is caused by parasitic blood-dwelling flukes in tropical and subtropical areas; about 200 million people are infected in 74 developing countries and territories [1,2]. Schistosomiasis is considered a neglected disease. It is caused by *Schistosoma japonicum* in China and Southeast Asia, *Schistosoma mansoni* in Africa and *Schistosoma haematobiumi* in the Middle East and Africa. In China, *S*. *japonicum* is distributed in some lake-marsh regions and mountain areas south of the Yangzi River, where around 330,000 people are infected from an at-risk population of 40 million [3]. Schistosomiasis threatens human health and causes great economic loss because it limits the breeding of bovines and other domestic animals in schistosomiasis-endemic areas. In addition to humans, more than 40 other mammals are reservoir hosts of *S*. *japonicum* in China [4]. Among the hosts, water buffaloes and yellow cattle are considered to the most important transmission source for schistosomiasis. The control of bovine schistosomiasis is critical for reducing disease prevalence in China. The development of simple, rapid, sensitive and specific diagnostic methods for animal surveillance is an urgent need for schistosomiasis control programs.

The conventional diagnostic methods for schistosomiasis are direct parasitological observations including observation of fecal eggs and miracidium hatching [5]. Although these methods are the gold standard for detecting animal schistosome infection, they are labor intensive and time consuming and not suitable for large-scale disease surveillance. The sensitivity of the parasitological diagnostic techniques depends on the rate of egg excretion and a major disadvantage is that the techniques have low sensitivity in low-prevalence endemic areas, resulting in high false-negative rates [6]. Molecular detection based on polymerase chain reaction (PCR) techniques is performed on urine, stool, or organ biopsy samples to detect DNA released from eggs [7,8]. Although these techniques are highly sensitive and specific, they are costly and require expensive equipment [9,10]. Recently, loop-mediated isothermal amplification assays have been used to amplify *S*. *japonicum* DNA and this method does not use expensive equipment. However, its use for diagnosis in endemic areas requires further system development and validation [11]. Serological methods such as circumoval precipitin test (COPT), indirect hemagglutination assay (IHA) and enzyme-linked immunosorbent assay (ELISA), are used to detect schistosome antigens or specific antibodies against them [12–14]. These methods are more sensitive and less time consuming than parasitological detection and are used for disease surveillance, especially in low-infection areas. However, most of the currently available antibody-detection techniques used whole-schistosome crude extracts, for example soluble egg antigens (SEAs), or soluble worm antigens (SWAs) as diagnostic antigens and have low specificity and poor reproducibility. Some defined schistosome antigens and purified recombinant antigens have been used instead of crude schistosome extracts as diagnostic antigens. These include 23 kDa membrane protein, 31/32 kDa protein, 26-kDa glutathione-S-transferase (GST), signaling protein 14–3–3, fructose bisphosphate aldolase, and 22-kDa tegumental membrane-associated antigen [15–18]. The use of these antigens partially improves the specificity of detection and some antigens showed potential value for evaluating the efficacy of drug treatment, but tests with most of the antigens had lower sensitivity than tests with crude worm antigens. Most of the tested diagnostic antigens were identified from the TG of schistosomes in previous studies [19–21].

The schistosome TG is a dynamic syncytial layer that covers the entire parasite and is composed of the surface membrane, the basal lamina and the matrix. The TG constitutes the major interface between the parasite and its host. Some TG proteins are key molecules that induce the host immune response against infection. The identification and characterization of host immune response target molecules in or exposed on the schistosome TG is crucial for screening diagnostic antigens.

Immunoproteomics is an increasingly popular method for identifying immunoreactive proteins from bacteria and parasites [22–29]. This technique has been used to identify schistosome proteins specifically recognized by schistosome-infected human or animal sera and some proteins can also induce short-lived antibody responses after praziquantel treatment. For example, Mutapi et al. reported that adult *S*. *haematobium* SWAs were recognized by pooled serum samples from infected Zimbabweans using immunoproteomics [22]. Pretreatment serum samples recognized 59 spots representing 21 proteins; serum samples after praziquantel treatment recognized an additional 12 spots representing 8 different proteins. However, only five proteins were recognized exclusively by posttreatment serum samples. Sera from infected rabbits have been used to identify antigenic proteins from adult *S*. *japonicum* worms [23]. Four immunoreactive antigens have been identified, and recombinant leucine aminopeptidase and fructose bisphosphate aldolase are candidate antigens for schistosomiasis japonicum diagnosis. Immune cross-reactivity between trematodes of *S*. *haematobium*, *S*. *bovis*, and *Echinostoma caproni* has been studied [24], and common cross-species antigens and species-specific targets have been identified. Excretory and secretory antigens of *S*. *japonicum* have been analyzed by immunoproteomic methods probing with sera from uninfected rabbits and rabbits 2- to 6-weeks after infection and 4–16 weeks after treatment. Investigation of antibody responses to schistosomiasis identified glyceraldehyde-3-phosphate dehydrogenase (GAPDH) as a major antigen inducing a short-lived antibody response [29]. These findings suggested immunoproteomics as a promising way to screen for novel serological diagnostic markers.

In this study, we used an immunoproteomics approach to identify potential immune diagnostic molecules from *S*. *japonicum* TG proteins extracted from adult schistosomes. Probes were derived from sera collected from uninfected rabbits and rabbits at 2 and 6 weeks after infection with *S*. *japonicum* as well as praziquantel-treated animals. The potential of schistosome recombinant proteins SjPGM and SjRAD23 as diagnostic antigens for water buffalo schistosomiasis was evaluated. This study provides information for screening potential diagnostic antigens and establishing more sensitive schistosomiasis diagnosis techniques.

## Methods

### Ethics statement

All animal care procedures were conducted in strict accordance to the Regulations for the Administration of Affairs Concerning Experimental Animals (1988.11.1), and all efforts were made to minimize suffering. All animal procedures were approved by the Animal Care and Use Committee of Shanghai Veterinary Research Institute, Chinese Academy of Agricultural Sciences for the use of laboratory animals (Permit ID Number: SHVRI 2011–0909).

### Worm collection

Cercariae of *S*. *japonicum* (a Chinese mainland strain) were shed from infected *Oncomelania hupensis* snails and numbers and viability were determined by observation with a light microscope. Fifteen New Zealand rabbits (male, 2.5–3.0 kg each) were infected with around 1000 cercariae through shaved abdominal skin [30]. Schistosomes were obtained by perfusion from the hepatic portal system and mesenteric veins of infected New Zealand rabbits at six weeks post infection [31]. Worms were manually washed five times in phosphate buffered saline (PBS, pH 7.4) at 37°C. Dead or damaged parasites were detected by microscopy and discarded. Intact parasites were used for further study. Animal care and all experimental procedures were according to the principles for the Care and Use of Laboratory Animals of the Shanghai Veterinary Research Institute, Chinese Academy of Agricultural Sciences.

### TG proteins and SEA preparation

TG proteins were obtained from freshly-obtained adult worms according to the method of Ricardo [32]. Parasites were rinsed five times with cold Tris-buffered saline (TBS, pH 7.4) and TG proteins were extracted with 1% Triton X-100 in TBS plus protease inhibitor cocktail (Roche, Germany) for 30 min with shaking at 4°C. The extract was centrifuged at 12,000 rpm for 45 min at 4°C, and the supernatant was collected for soluble TG proteins, which were concentrated by suspension in cold acetone at 4°C for 2 h and centrifuged at 12,000 rpm for 1 h at 4°C. Pellets were resuspended in lysis buffer containing 7 M urea, 2 M thiourea, 65 mM Tris, 2% dithiotreitol (DTT), 4% 3-([3-cholamidopropyl] dimethyl-ammonio)-1-propanesulfonate (CHAPS), 0.2% IPG buffer and 0.1% (v/v) protease inhibitor mixture (Merck, Germany), and centrifuged at 12,000 rpm for 40 min at 4°C to remove insoluble material. SEA was prepared as described in previous literature [33,34]. Protein concentrations of TG extracts and SEA were measured by Bradford assay (Bio-Rad, USA). Samples were stored at -80°C.

### Serum samples

Rabbit sera: Serum samples were obtained from 15 rabbits infected with 100 ± 2 *S*. *japonicum* cercariae. At 6 weeks post infection, all fecal samples from rabbits were examined by the miracidia hatching technique [35,36]. All miracidia-positive rabbits were treated with praziquantel (100 mg/kg of body weight; Nanjing Pharmaceutical Factory, China) and no miracidia were found one month after praziquantel treatment by the miracidia hatching technique. Sera were collected before infection, at 2 and 6 weeks post-cercariae challenge, and monthly after praziquantel treatment for 8 months. At the end of the experiment no schistosome worms were found after perfusing from the hepatic portal system and mesenteric veins of the rabbits.

Buffalo sera: Serum samples from 104 schistosomiasis-positive buffaloes, confirmed by the miracidia-hatching method [35,36], were obtained from Anhui Province, an area of endemic schistosomiasis. Sera from 14 buffaloes infected with paramphistomum and 9 infected with *Fasciola gigantica* were collected as well as 60 normal serum samples from healthy buffaloes in Henan Province, where schistosomiasis is not endemic. Serum samples were stored at -80°C.

### Two-dimensional electrophoresis

Two sets of two-dimensional electrophoresis (2-DE) separations were conducted in parallel: one with 800 μg of TG for protein identification and the other with 300 μg of TG for Western blotting. In first-dimensional electrophoresis, samples were mixed with an equal volume of rehydration solution (8 M urea, 4% CHAPS, 2% DTT and 2% IPG buffer pH 3–10), and pooled protein samples were applied to isoelectric focusing in 13 cm IPG strips with a nonlinear pH range of 3–10 (GE Healthcare, USA). After in-gel rehydration at 30 V, room temperature for 12 h, isoelectric focusing was conducted using an Ettan II IPG-phor apparatus (GE Healthcare, USA) for 1 h at 500 V, 1 h at 1000 V, and 10 h at 8000 V. Strips were reduced in equilibration buffer (6 M urea, 0.05 M Tris-HCL, pH 6.8, 2% SDS, 30% glycerol and trace bromophenol blue) containing 1% DTT for 15 min and alkylated in equilibration buffer containing 2.5% DTT for 15 min. In second-dimension electrophoresis, equilibrated strips were subjected to SDS-PAGE in 12% polyacrylamide 13 cm gels and Tris-glycine-SDS buffer (25 mM Tris, pH 8.3,192 mM glycine, 0.1% SDS) at 15 mA for 20 min and 30 mA until the dye front reached the bottom of the gel using a Hoefer SE600 system (GE Healthcare, USA). After separation, proteins were visualized by Coomassie brilliant blue G250 (CBB, Invitrogen, USA) or transferred to PVDF membranes (GE Healthcare, UK) for immunoblots.

### Immunoblots

Proteins were transferred to PVDF membranes using a semidry system (TE77, Amersham Pharmacia Biotech, UK) in 39 mM glycine, 48 mM Tris base and 20% methanol at 175 mA for 2 h 15 min. To check transfer efficiency, membrane-bound proteins were detected with a reversible staining kit (Sangon Biotech Co., Ltd., China) according to the manufacturer’s instructions. Blots were blocked with 5% skim milk in PBS containing 0.05% Tween-20 (PBST) at room temperature for 2 h and washed three times for 10 min with PBST. Membranes were incubated with rabbit sera from uninfected rabbits or rabbits at 2 or 6 weeks after-schistosome infection, or 1, 2, 3, 4, 5, 6, 7, or 8 months after treatment with praziquantel. The sera were for diluted 1:50 in PBST and incubations were overnight at 4°C. After three washes, membranes were incubated with goat anti-rabbit IgG conjugated to horseradish peroxidase (HRP, Beyotime, China) diluted 1:1,000 in PBST for 1 h at room temperature. After washing with PBST, membranes were visualized using an enhanced 3, 3′-diaminobenzidine substrate kit (Tiangen Biotech, China). Immunoblots were repeated three times. Images from immunoblots and parallel stained gels were visualized with a VersaDoc Imaging System Model 4000 (Bio-Rad, USA) using Quantity One software Version 4.5.1 (Bio-Rad, USA) and analyzed by the PDQuest version 7.3.0 software (Bio-Rad, USA).

### In-gel tryptic digestion and protein analysis by MALDI-TOF/TOF-MS

Gel spots containing matched immunogenic proteins of interest were excised manually from stained gels and subjected to in-gel trypsin digestion as follows: 1 mm^3^ gel plugs were destained in 100 mmol/L NH_4_HCO_3_/30% ACN for 20 min. After lyophilization, samples were digested in 100 mmol/L NH_4_HCO_3_ containing 12.5 ng/μL trypsin at 37°C overnight. Tryptic peptides were extracted three times with 50% ACN /0.1% TFA and dried by centrifugal lyophilization.

Peptide samples were sent to Shanghai Applied Protein Technology Co. for MALDI-TOF/TOF MS analysis. Data for protein identification were obtained using a MALDI-TOF-TOF instrument (4800 proteomics analyzer; Applied Biosystems). Combined peptide mass fingerprinting and MS/MS queries used the Mascot search engine 2.2 (Matrix Science, UK) against the NCBI schistosome sequence database and the NCBI metazoa sequence database with parameter settings: confidence interval 95%, trypsin digestion, fixed modification of carbamidomethyl (C), variable modifications of oxidation (M), one max missed cleavages, 100 ppm mass accuracy, peptide mass tolerance ± 0.4 Da and peptide charge state 1+. Protein score C.I.% ≥ 95% were considered significant for manual validation.

### Expression and purification of recombinant SjPGM and SjRAD23

The cDNA fragment encoding SjPGM (GenBank accession no. FN315287) was amplified by PCR with forward primer (5’- ATAGGATCCATGGCTCCCTAC-3’) and reverse primer (5’-GCGCTCGAGTTATTTCTTCTTT-3’) with *Bam*HI and *Xho*I endonuclease sites underlined. According to the sequence of SjRAD23 (GenBank accession no. FN314619) forward and reverse primers 5’-GCGGGATCCATGAAGGTTACTTTC-3’ and 5’- CAGCTCGAGTCATACCATTTCATC-3’ with the same endonuclease sites underlined were designed to amplify the cDNA. PCR amplification used cDNA from *S*. *japonicum* adult worms as templates. Products contained complete open reading frames (ORFs) for SjPGM and SjRAD23 were inserted into the expression vector pET28a(+) (Novagen, USA). Plasmids pET28a(+)-SjPGM and pET28a(+)-SjRAD23 were confirmed by sequencing and transformed into *Escherichia coli* BL21 (DE3) (Beijing TransGen Biotech, China). Positive transformants were cultured in Luria-Bertani broth containing kanamycin (1 mM) and isopropyl-β-D-thiogalactopyranoside (final concentration 1 mM) was added to express rSjPGM and rSjRAD23. Purification of recombinant proteins was by His•Bind Resin chromatography (Novagen, USA) following the manufacturer’s instruction.

### Immunolocalization of SjPGM and SjRAD23 in adult *S*. *japonicum* worms

BALB/c mice were subcutaneously injected with 20 μg of purified rSjPGM or rSjRAD23 emulsified in 206 adjuvant respectively according to a previously-described method [37]. PBS was used as the negative control and sera were collected two weeks after the last immunization. Frozen sections (6 μm) of adult worms were fixed in acetone for 10 min at -20°C. After blocking with 10% goat serum in PBST for 2 h at room temperature, slices were incubated with mouse serum against rSjPGM, rSjRAD23 or naïve mouse serum diluted 1:100 in PBST overnight at 4°C and probed with CY3-conjugated goat anti-mouse IgG (Beyotime, China), diluted 1:1,000 in PBST for 1 h at room temperature. Parasite nuclei were stained with 2-(4-amidinophenyl)-6-indolecarbamidine dihydrochloride at a final concentration of 10 μg/mL for 5 min at room temperature and visualized by fluorescence microscopy (Nikon, Japan). Sections were washed 3 times for 5 min with PBST between steps.

### Evaluation of rSjPGM and rSjRAD23 as diagnostic antigens for *S*. *japonicum*

Specific IgG antibody against rSjPGM and rSjRAD23 and SEA in sera from five rabbits as described above was detected by ELISA. Protocol optimization and dilution of testing reagents was determined by checkerboard titration analysis. Microtiter plates (Costar, USA) were coated with 0.5 μg rSjPGM, 1.5 μg rSjRAD23, 2 μg SEA diluted with 100 μL carbonate bicarbonate buffer (pH 9.6) in each well and incubated at 4°C overnight. After blocking with 3% bovine serum albumin in PBST, all tested serum samples were diluted 1:100 with PBST and incubated for 1 h at 37°C. HRP-conjugated goat anti-rabbit immunoglobulin G (CWBIO, China) was added at 1:5000 for rSjPGM, 1:10000 for rSjRAD23 and 1:1000 for SEA in PBST. Washes were with PBST, 3 times for 10 min between steps. After incubation for 1 h at 37°C, substrate 3, 3&rsquo;5, 5′-tetramethyl benzidine dihydrochloride was added (100 μL/well) and reactions stopped with 2 M sulfuric acid (50 μL/well). Absorbance was measured at 450 nm using a microplate ELISA reader (BioTek, USA). Equal volumes of sera from thirty uninfected rabbits were pooled and used as negative control. The cutoff value for ELISA was 2.1 times the mean absorbance value of negative control.

### Diagnosis of water buffalo schistosomiasis using rSjPGM and rSjRAD23 as detecting antigens

Sera from 187 water buffaloes including 104 naturally infected with *S*. *japonicum*, 14 infected with paramphistomum, 9 infected with *F*. *gigantica*, and 60 uninfected were examined by ELISA using rSjPGM, rSjRAD23 and SEA as diagnostic antigens. Based on checkerboard titration analysis, microtiter plates were coated overnight at 4°C with 100 μL per well 5 μg/mL rSjPGM, 15 μg/mL rSjRAD23 or 15 μg/mL SEA diluted in carbonate bicarbonate buffer (pH 9.6). Plates were blocked with 1% gelatin for 1 h at 37°C (150 μL/well). All tested sera samples were diluted 1:100 with PBST (100 μL/well) and incubated for 1 h at 37°C. HRP-conjugated rabbit anti-bovine immunoglobulin G diluted 1:3000 in PBST was added to wells (100 μL/well). PBST washes were 3 times, 10 min between step, 100 μL 3,3&rsquo;5, 5′-tetramethyl benzidine dihydrochloride was added to each well and reactions were stopped using 2 M sulfuric acid (50 μL/well). Optical densities were read at 450 nm using a microplate reader (BioTek, USA). Equal volumes of sera from thirty uninfected water buffaloes were pooled and used as the negative control. The cutoff value for ELISA was 2.1 times the mean absorbance value of the negative control. Sensitivity and specificity for rSjPGM- ELISA and rSjRAD23- ELISA were compared with SEA-ELISA.

### Statistical analyses

Statistical analysis was performed using Stata software (version 10.1/SE). Sensitivity and specificity were calculated as sensitivity = no. of true positives/(no. of true positive + no. of false negatives), and specificity = no. of true negatives/(no. of false positive + no. of true negatives). The 95% confidence intervals (CI) were determined for the sensitivity, specificity and crossreactivity of each test.

## Results

### 2-DE analysis of *S*. *japonicum* TG proteins

**Fig 1:**
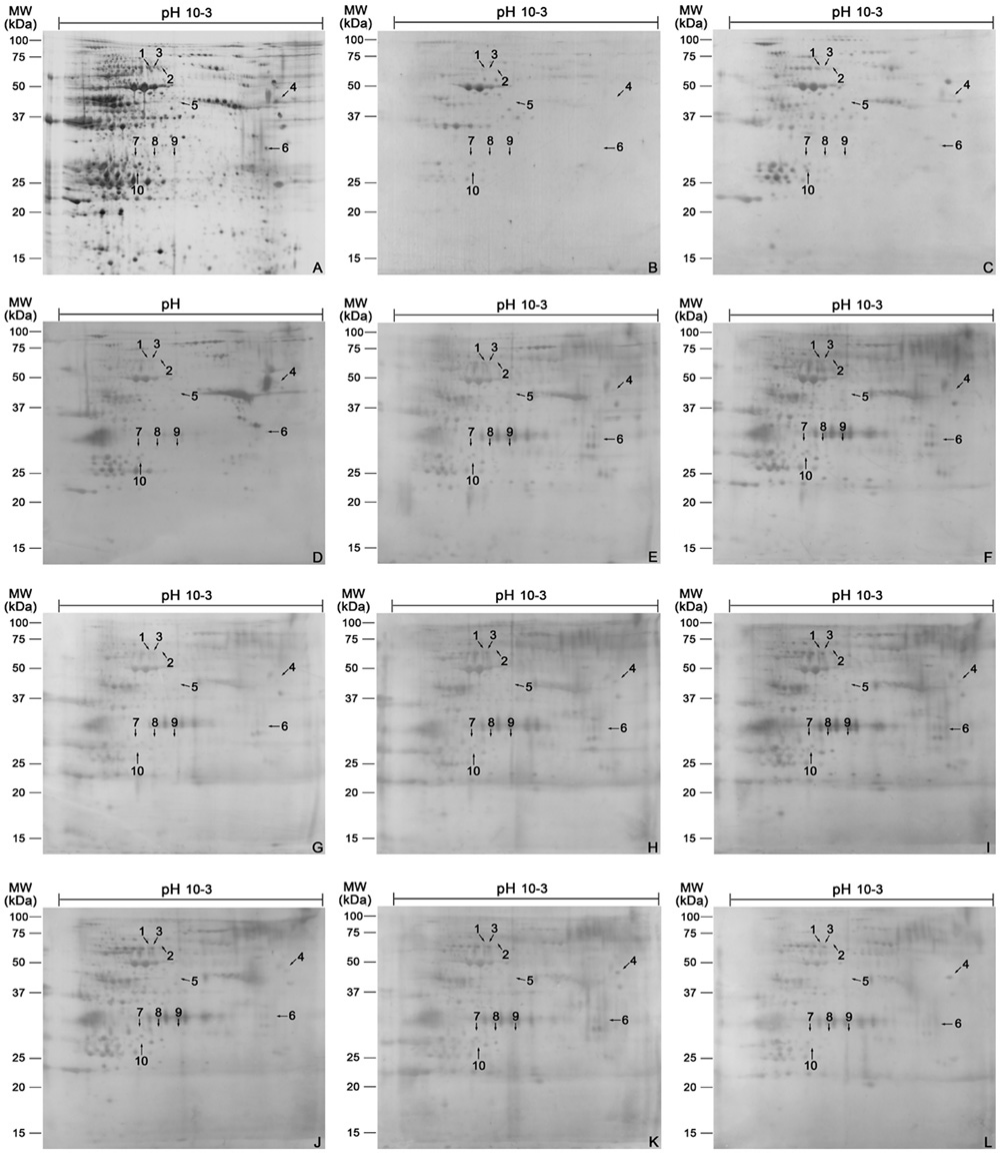
2-DE analysis and immunoblot patterns using TG proteins of adult *S*. *japonicum* as antigens and sera collected from rabbits at stages of uninfected, infected with *S. japonicum*, and post-treatment of praziquantel as the primary antibodies. (A) 2-DE separation of TG proteins on CBB stained gel over the pH range 10–3. (B) Immunoproteomics using sera of uninfected rabbit. (C-D) Immunoproteomics using sera of rabbits at 2 (C) and 6 (D) weeks post-infection. (E-L) Immunoproteomics using sera of rabbits at 1 (E), 2 (F), 3 (G), 4 (H), 5 (I), 6 (J), 7 (K) and 8 (L) months post-treatment.

More than three batches of TG proteins extracted from adult *S*. *japonicum* were pooled and separated on 2-DE gels (Fig. 1A) and spots were visualized. After analysis by PDQuest software, 2232 putative spots were detected with molecular weights (MW) ranging from 15 to 100 kDa and isoelectric point (p*I*) from 3 to 10. Around 1500 proteins were MW 20–75 kDa and p*I* 4–9.

### Immunoblot analysis of TG proteins

Comparison of sera from noninfected rabbits and rabbits at 2 and 6 weeks post-infection and 1–8 month post-treatment was conducted to screen for possible diagnostic candidates. After incubating proteins on membranes with pooled serum sample, 182 protein spots reacted with the noninfected sera. The serum samples from 2 and 6 weeks post-infection rabbits detected 320 and 296 spots, respectively. More positive spots were detected by sera from rabbits at 1 and 2 months after treatment. Sera from rabbits 2 months after treatment recognized the most spots, at 416. The number of spots recognized by serum samples from rabbits at 3–8 month after treatment was reduced, with 8-month posttreatment serum detecting only 265 spots. Ten protein spots were not recognized by uninfected sera (Fig. 1B, Table 1) but reacted with sera from rabbits at 2 and 6 weeks after *S*. *japonicum* infection (Fig. 1C-D, Table 1). These 10 spots were not detected again early after praziquantel treatment began (Fig. 1E-L, Table 1). These 10 spots represented 6 unique proteins, as identified by MALDI-TOF/TOF-MS (Table 2). Of these, *S*. *japonicum* phosphoglycerate mutase (SjPGM) and UV excision repair protein RAD23 homolog B (SjRAD23) were recognized by serum from infected but not uninfected or drug-treated rabbits and were used for the following study.

**Table 1:** Identification of TG proteins which may be potential diagnostic antigens for schistosomiasis japonicum (-, negative/disappearance; +, positive/appearance).

Spot ID	Rabbit uninfected sera	Rabbit sera at 2 and 6 weeks after infection	Rabbit sera at different months after treatment of praziquantel							
			1	2	3	4	5	6	7	8
1	-	+	+	+	-	-	-	-	-	-
2	-	+	+	+	+	-	-	-	-	-
3	-	+	+	+	+	-	-	-	-	-
4	-	+	-	-	-	-	-	-	-	-
5	-	+	+	-	-	-	-	-	-	-
6	-	+	+	+	-	-	-	-	-	-
7	-	+	-	-	-	-	-	-	-	-
8	-	+	-	-	-	-	-	-	-	-
9	-	+	-	-	-	-	-	-	-	-
10	-	+	-	-	-	-	-	-	-	-

**Table 2:** MALDI-TOF-TOF MS identification of some TG proteins which may be potential diagnostic antigens for schistosomiasis japonicum.

Spot ID	Accession no.	Description	Theoretical Mr/pI	Protein score	Protein score C.I.%	Sequence coverage (%)^*a*^	No. of peptides matched
1–3	gi|56752663	Putative heat shock protein	52487/5.91	395	100	29.98	12
4	gi|226470142	UV excision repair protein RAD23 homolog B (RAD23)	37914/4.46	82	99.969	34.00	7
5	gi|29841387	similar to GenBank Accession Number J04017 heat shock protein 86 in Schistosoma mansoni	31154/6.77	92	99.997	27.27	5
6	gi|226489240	Charged multivesicular body protein 5	24997/4.63	475	100	25.89	6
7–9	gi|226489007	similar ot Metallo-beta-lactamase domain-containing protein 1	26537/4.84	324	100	43.64	8
10	gi|226472666	phosphoglycerate mutase (PGM)	28474/6.32	529	100	52.80	11

^*a*^ The percentage coverage was defined as the ratio (%) of the protein sequence covered by the matched peptides.

### Expression and purification of rSjPGM and rSjRAD23

**Fig 2:**
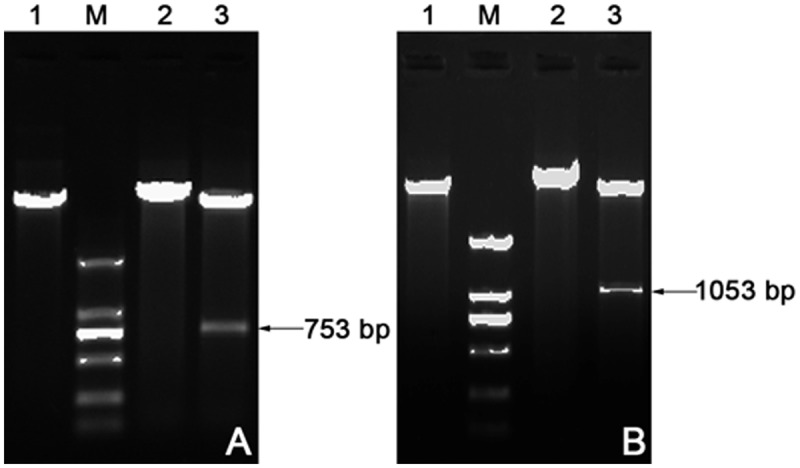
The recombinant plasmid pET28a(+)–SjPGM (A) and pET28a(+)–SjRAD23 (B) were identified by enzyme digestion. Lane 1: The blank plasmid pET28a(+). Lane 2: The recombinant plasmid pET28a(+)–SjPGM or pET28a(+)–SjRAD23. Lane 3: The recombinant plasmid pET28a(+)–SjPGM and pET28a(+)–SjRAD23 digested with *Bam*HI and *Xho*I restriction enzymes. Lane M: Marker DL2000 DNA Ladder.

**Fig 3:**
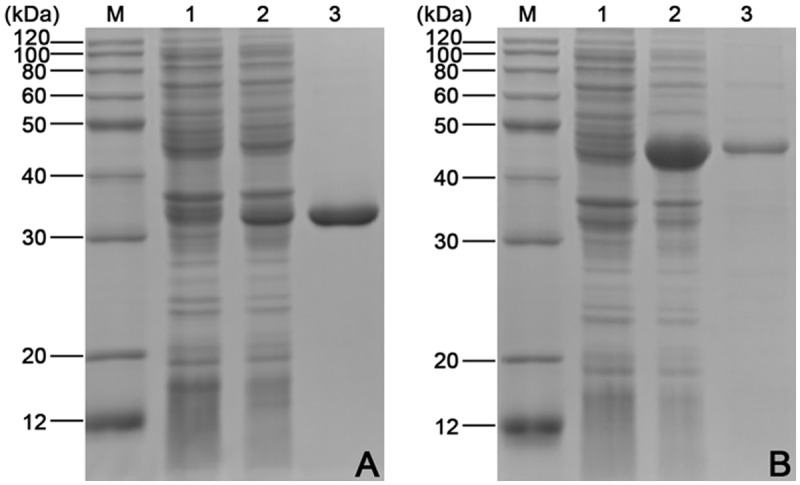
Expression and purification analysis of rSjPGM (A) or rSjRAD23 (B) protein using SDS-PAGE (12%). Lane M: Molecular mass markers. Lane 1: Total extract from pET28a(+) after induction with 1 mM IPTG. Lane 2: Total extract from rSjPGM or rSjRAD23 after induction with 1 mM IPTG. Lane 3: rSjPGM or rSjRAD23 purified through Ni^2+^-charged column chromatography.

To evaluate the potential of rSjPGM and rSjRAD23 as schistosomiasis diagnostic antigens, the ORFs encoding the 753 bp SjPGM and 1053 bp SjRAD23 were amplified, sequenced (Fig. 2) and cloned into expression vectors. Soluble His-tagged fusion protein with expected sizes of 32 kDa for rSjPGM and 44 kDa for rSjRAD23 were purified by affinity chromatography and purity was analyzed by SDS—PAGE (Fig. 3).

### Immunolocalization of SjPGM and SjRAD23 in schistosomes

**Fig 4:**
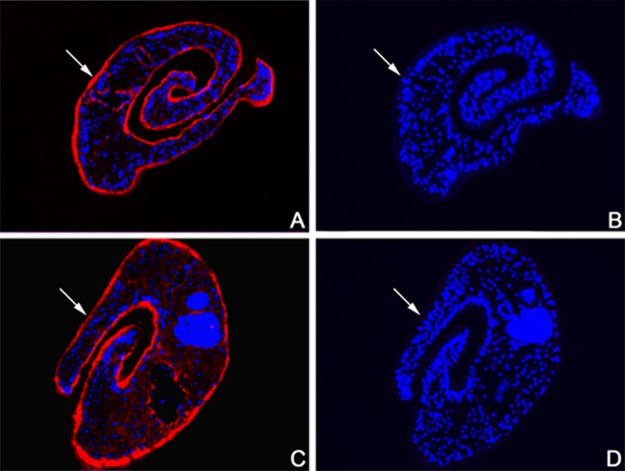
Immunolocalization of SjPGM and SjRAD23 in adult worm of *S*.*japonicum*. The sections were probed with anti-rSjPGM mice serum (A), anti-rSjRAD23 mice serum (C) and naive mice serum (B, D).

Immunofluorescence assays were used to determine the distribution of SjPGM and SjRAD23 in 42-day-old schistosome adult worms using anti-rSjPGM and anti-rSjRAD23 from immunized mice and naïve mouse serum (as a negative control). Compared with the negative control, specific staining was observed in sections probed with rSjPGM-specific and rSjRAD23-specific sera, but not in negative control samples, indicating the two TG-associated antigens were mainly present on the surface of adult *S*. *japonicum* (Fig. 4).

### Evaluation of rSjPGM and rSjRAD23 as diagnostic antigens for *S*. *japonicum*

**Fig 5:**
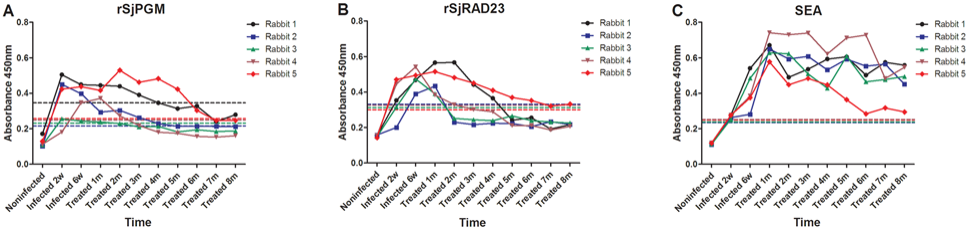
Antibody profiles as OD values using rSjPGM (A), rSjRAD23 (B) and SEA (C) as antigens and sera collected from rabbits at stages of uninfected, infected with *S. japonicum*, and post-treatment of praziquantel as the primary antibodies.

Sera were collected from 5 rabbits before infection, 2 and 6 weeks after infection and between 1 and 8 months after treatment with praziquantel. The sera were used in ELISAs with rSjPGM, rSjRAD23 or SEA (control) as diagnostic antigens (Fig. 5). Specific IgG antibodies against the three antigens were detected in sera from all rabbits at 0, 2 and 6 weeks post-infection, and each month after praziquantel treatment. By rSjPGM-ELISA, one rabbit showed seroconversion in each month from months 2 to 7, except for month 6. All five rabbits (5/5) were schistosomiasis-negative at 7 months after treatment. By rSjRAD23-ELISA, two rabbits at month 2, one at month 3 and one at month 5 (4/5) showed seroconversion. By SEA-ELISA, five serum samples (5/5) remained schistosomiasis-positive from months 1 to 8 after treatment.

### Diagnosis of water buffalo schistosomiasis using rSjPGM and rSjRAD23 as detecting antigens

**Fig 6:**
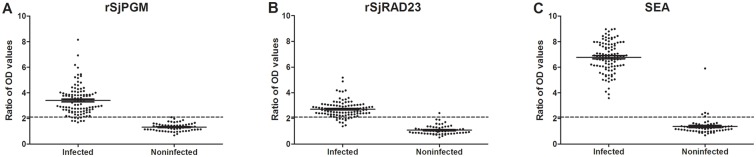
ELISA analysis of rSjPGM (A), rSjRAD23 (B) and SEA (C) for diagnosis of buffaloes schistosomiasis. Sera from 104 schistosome infected buffaloes and 60 noninfected buffaloes.

To assess the potential of rSjPGM and rSjRAD23 as diagnostic antigens for water buffalo schistosomiasis, sera from 104 schistosome-infected water buffaloes, 60 noninfected buffaloes and 23 water buffaloes infected with other trematodes were tested using rSjPGM, rSjRAD23 or SEA (control) as detecting antigen, yielding a sensitivity for rSjPGM-ELISA of 91.35% (95/104, 95% CI: 84.21–95.97%) and for rSjRAD23-ELISA of 88.46% (92/104, 95% CI: 80.71–93.89%). These numbers were lower than for SEA-ELISA 100.00% (104/104, one-sided, 97.5% CI: 96.52–100%). The specificity of rSjPGM-ELISA was 100.00% (60/60, one-sided, 97.5% CI: 94.04–100.0%) and rSjRAD23-ELISA was 98.33% (59/60, 95% CI: 91.06–99.96%), which was higher than SEA-ELISA at 91.67% (55/60, 95% CI: 81.61–97.24%) (Figs. 6, S1, Table 3). The three antigens were also used to detect serum from paramphistomum-exposed or *F*. *gigantica*-exposed buffaloes. False-positive rates were 7.14% (1/14, 95% CI: 0.18–33.87%) and 11.11% (1/9, 95% CI: 0.28–48.25%) for rSjPGM-ELISA; 14.29% (2/14, 95% CI: 1.78–42.81%) and 0.00% (0/9, one-sided, 97.5% CI: 0–33.63%) for rSjRAD23-ELISA; and 50.00% (7/14, 95% CI: 23.04–76.96%) and 44.44% (4/9, 95% CI: 13.70–78.80%) for SEA-ELISA (Table 3).

**Table 3:** Comparison of positive rates of antibodies against rSjPGM, rSjRAD23 and SEA detected in buffaloes sera by ELISA.

Sera samples	No. of cases	rSjPGM		rSjRAD23		SEA	
		No. of positive	Positive rate (%)	No. of positive	Positive rate (%)	No. of positive	Positive rate (%)
Uninfected buffaloes	60	0	0.00	1	1.67	5	8.33
*S*. *japonicum*-infected buffaloes	104	95	91.35	92	88.46	104	100.00
Paramphistomum-infected buffaloes	14	1	7.14	2	14.29	7	50.00
*F*. *gigantica*-infected buffaloes	9	1	11.11	0	0.00	4	44.44

*Note*: Sensitivity = no. of true positives/(no. of true positive + no. of false negatives), and specificity = no. of true negatives/(no. of false positive + no. of true negatives.

## Discussion

The absence of information on the role of animal hosts in the transmission of *S*. *japonicum* has hampered efforts to control this schistosome species. The lack of efficient, sensitive diagnostic tools for animal surveillance could be a factor in the infection and transmission of schistosomes among reservoir hosts [38]. In hosts, cercariae penetrate the skin and migrate to the hepatic portal system, where they mature and survive for several years. Schistosomes exposed to the host immune system stimulate a complex host immune response. Therefore, serum contains specific antibodies for schistosome antigens and antigens recognized by these antibodies could be potential diagnostic biomarkers for schistosomiasis. Generally, soluble crude antigens prepared from schistosome eggs or adult worms have been used as major antigens for immunodiagnosis [23,29]. However, these antigens are complex and have unsatisfactory specificity and high cross-reactivity with serum from animals infected with other parasitic helminths, such as *Fasciola hepatica* and *F*. *gigantica*. Cross-reactivity limits the application of SEA-ELISA since schistosome infection often overlaps with other helminths in endemic areas. Also, the use of currently used antigens cannot distinguish between current and past infection or be used to evaluate the effectiveness of drug treatment [39]. Eggs remain in livers or intestines of infected hosts after drug treatment and continue to stimulate the production of antibodies, so antibodies to crude extract antigens with thousands of schistosome proteins decline very slowly [40]. Most are present at a high level for months or even up to 1 or 2 years after treatment. Therefore, screening for more sensitive and specific biomarkers for diagnosis of animal schistosomiasis is urgent for disease control.

A comprehensive genome-wide or proteome-wide analysis of host immune responses to schistosome infection is essential for the identification of novel antigen targets for immunodiagnostic tests and future vaccine development [41]. Recently, Xu et al. identified the protein marker SjSP-13 by screening a GST-fusion protein array and showed that rSjSP13-ELISA diagnostic method had substantial advantages over currently used methods [42]. Wang et al., by immunoscreening excretory and secretory antigens of *S*. *japonicum*, identified SjGAPDH as a diagnostic antigen that could induce short-lived antibody responses and developed a sensitive and specific diagnostic method for schistosomiasis [29]. In this study, schistosome TG proteins were investigated by a differential immunoproteomics technique. Changes in immunoreactive proteins recognized by sera from uninfected rabbits, rabbits infected with schistosomes, and rabbits treated with praziquantel were compared and analyzed. TG proteins were chosen because they contain many kinds of antigens that are directly exposed to the host environment and induce the host immune response against schistosomes. Some of the proteins might be potential diagnostic markers for this disease. In this study, different sources of rabbit sera were used to identify proteins that were not recognized by sera from uninfected animals. Specific antibodies against these antigens might persist at a high level during infection but decline quickly after drug treatment. We hypothesized that the application of some of these proteins as diagnostic antigens might overcome problems in schistosomiasis serologic diagnosis such as low specificity, high cross-reactivity with other parasitic helminths and inability to be used for evaluation of drug effectiveness or to distinguish between current and past infection. Probing with pooled non-infected rabbit sera we detected 182 reactive spots. More reactive spots were observed by probing with sera from 2 and 6 weeks infected animals. We also found that positive spots appeared one and two months after praziquantel treatment and other positive spots recognized by sera from infected animals disappeared gradually when probed with sera from praziquantel-treated animals. False-positive spots were detected by probing with sera from uninfected animals, explaining why low specificity is often observed in immunodiagnostic tests using crude antigens. The novel positive spots detected after schistosome infection could be targets recognized by specific schistosome antibodies. Additional positive spots detected at the early stage of praziquantel treatment suggested that hidden worm antigens or epitopes were exposed and recognized by the host’ immune systems after drug treatment. After careful comparison, we chose 10 protein spots that were recognized by the sera of infected rabbits 2 and 6 weeks after challenge. These spots were negative when probed with sera from rabbits taken 4 months after treatment. These 10 protein spots were analyzed by MALDI-TOF/TOF MS and 6 proteins were identified. Some of the proteins had multiple isoforms with different p*I* and MW, which may represent post-translational modifications or cleavage events. Although the proteins were identified by two or more spots on immunoblots, they might share amino acid sequences or epitopes. SjPGM and SjRAD23, two schistosome TG proteins, were selected for further study.

PGM catalyzes the interconversion of 2- and 3-phosphoglycerate in the glycolytic and gluconeogenic pathways and is present in most organisms. PGM is required for the energy metabolism necessary for parasite activity and survival. PGM is found in the TG surface membranes of *S*. *japonicum* [43], *S*. *mansoni* [19] and *S*. *bovis* [20]. It is exposed to the host immune system and might participate in the complex host-parasite relationship. This protein was also recognized by sera from patients infected with *Schistosoma haematobium* [22] and *Brugia malayi* [44]. These results suggest that SjPGM could be a diagnostic marker for schistosomiasis. In eukaryotes, the nucleotide excision repair system acts by removing a range of structurally unrelated DNA lesions such as those induced by ultraviolet light or bulky DNA adducts [45,46]. In *S*. *mansoni*, the RAD23 gene is expressed in all developmental stages of the parasite: egg, sporocysts, cercariae, schistosomula and adult worms. This gene showed differential expression in the presence of DNA-damaging agents [47]. In this study, PGM and RAD23 were successfully subcloned, expressed as soluble proteins in *E*. *coli* and purified. Immunofluorescence showed that the two proteins were TG-associated and mainly distributed on the surface of adult *S*. *japonicum*.

In order to verify the reliability of the immunopoteomics screening, rSjPGM and rSjRAD23 were detected and evaluated by another method (ELISA) using 5 individual rabbits. Specific IgG antibodies to rSjPGM and rSjRAD23 in the sera of early (2 weeks) or acute (6 weeks) schistosome-infected rabbits increased in 5 tested animals, then declined gradually from 2 months after praziquantel treatment. However, the level of specific IgG antibody to SEA had not changed significantly even after 8 months after treatment. This result was quite consistent with immunoproteomics screening and suggested that these two proteins could induce a short-lived antibody response. Unfortunately, no water buffaloes’ sera collected monthly after praziquentel treatment were available to help validate this result. The potential of the two recombinant proteins to evaluate the effectiveness of chemotherapy against *S*. *japonicum* in water buffaloes thus needs to be further studied in the future.

The diagnostic potential of rSjPGM and rSjRAD23 for schistosomiasis in water buffaloes was evaluated. Our data indicated that the sensitivity of rSjPGM-ELISA and rSjRAD23-ELISA were slightly lower than the sensitivity of SEA-ELISA. Besides sensitivity, specificity and cross-reactivity to other pathogens are also important in serological diagnosis. On the limitation of lacking sera from buffaloes infected with some other parasites, only 14 buffaloes infected with paramphistomum and 9 infected with *Fasciola gigantica* were used to detect the cross-reactivity of the two antigens. The results showed that the specificity of rSjPGM-ELISA and rSjRAD23-ELISA was clearly higher than SEA-ELISA, and cross-reactivity of them was clearly lower than SEA-ELISA. Taken together, the results obtained for rSjPGM-ELISA and rSjRAD23-ELISA showed that the schistosomiasis diagnosis techniques developed in this study have substantial advantages over SEA-ELISA. In this study, between the two newly developed methods, rSjPGM-ELISA was more sensitive and specific than rSjRAD23-ELISA.

In order to further confirm the diagnostic effect of the two antigens, more sera from *S*. *japonicum*-infected buffaloes, uninfected buffaloes, and some other parasites infected buffaloes should be analyzed in future studies. Especially the sera from buffaloes infected with some other flukes, such as *Fasciola hepatica*, *Orientobilharzia turkestanicum* etc., should be tested to further evaluate the crossreactivity. At the same time, the two recombinant antigens should be validated using infected water buffaloes’ sera after praziquantel treatment to evaluate the potential value for the effectiveness of drug treatments and for distinguishing between current and past infection.

In summary, this study showed that immunoproteomics was a promising method for screening for immunodiagnostic antigens for schistosomiasis. The two schistosome TG proteins SjPGM and SjRAD23 were identified as potential diagnostic markers for the disease. High sensitivity and specificity and low cross-reactivity were observed for rSjPGM-ELISA and rSjRAD23-ELISA, which were used to detect water buffalo schistosomiasis. Antibodies to rSjPGM and rSjRAD23 are probably short-lived since they declined quickly after chemotherapy. Therefore, the two recombinant proteins have the potential to evaluate the effectiveness of drug treatments and for distinguishing between current and past infection, and this needs to be further studied in water buffaloes in the future studies.

## Supporting Information

S1 Checklist STARD checklist. (DOC)

S1 Fig Flowchart used for studies of diagnostic tests. (TIF)
