# Supplementary figures and images for: Screening Diagnostic Candidates for Schistosomiasis from Tegument Proteins of Adult Schistosoma japonicum Using an Immunoproteomic Approach
Source: PLoS Negl Trop Dis. 2015 Feb 23;9(2):e0003454. doi: 10.1371/journal.pntd.0003454 (PMC4338221; doi:10.1371/journal.pntd.0003454)

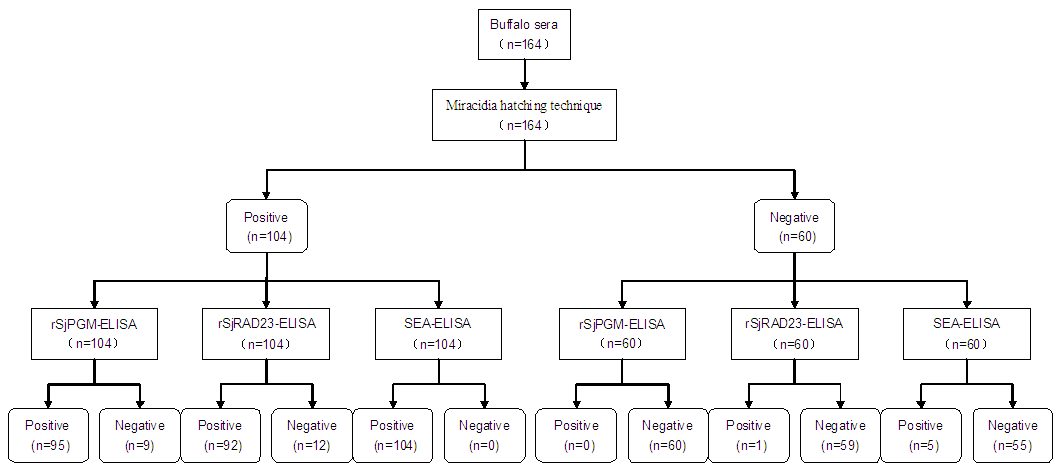

Supplement: S1 Fig — (TIF) [file pntd.0003454.s002.tif]
